# Pseudo-atomic orbital behavior in graphene nanoribbons with four-membered rings

**DOI:** 10.1126/sciadv.abl5892

**Published:** 2021-12-22

**Authors:** Peter H. Jacobse, Zexin Jin, Jingwei Jiang, Samuel Peurifoy, Ziqin Yue, Ziyi Wang, Daniel J. Rizzo, Steven G. Louie, Colin Nuckolls, Michael F. Crommie

**Affiliations:** 1 Department of Physics, University of California, Berkeley, CA 94720, USA.; 2 Department of Chemistry, Columbia University, New York, NY 10027, USA.; 3 Department of Physics, Columbia University, New York, NY 10027, USA.; 4 Materials Sciences Division, Lawrence Berkeley National Laboratory, Berkeley, CA 94720, USA.; 5 Kavli Energy NanoSciences Institute at the University of California, Berkeley and the Lawrence Berkeley National Laboratory, Berkeley, CA 94720, USA.

## Abstract

The incorporation of nonhexagonal rings into graphene nanoribbons (GNRs) is an effective strategy for engineering localized electronic states, bandgaps, and magnetic properties. Here, we demonstrate the successful synthesis of nanoribbons having four-membered ring (cyclobutadienoid) linkages by using an on-surface synthesis approach involving direct contact transfer of coronene-type precursors followed by thermally assisted [2 + 2] cycloaddition. The resulting coronene-cyclobutadienoid nanoribbons feature a narrow 600-meV bandgap and novel electronic frontier states that can be interpreted as linear chains of effective p*_x_* and p*_y_* pseudo-atomic orbitals. We show that these states give rise to exceptional physical properties, such as a rigid indirect energy gap. This provides a previously unexplored strategy for constructing narrow gap GNRs via modification of precursor molecules whose function is to modulate the coupling between adjacent four-membered ring states.

## Teaser

A type of nanoribbon with four-membered rings was synthesized and was found to behave like a hologram of atomic orbitals.

## INTRODUCTION

The prospect of using graphene nanoribbons (GNRs) as next-generation nanoelectronic components has strengthened since their first bottom-up synthesis just over a decade ago (*1*, *2*). Much excitement has been generated by their expected conductance properties as well as the tremendous control over nanoribbon geometry provided by chemical bottom-up methodologies and the resulting unparalleled tunability of their electronic structure. Early research largely focused on the potential role of GNRs in field-effect transistors (*3*, *4*), revolving around tailoring the size of their bandgap through width, edge, and heteroatom engineering (*2*, *5*–*7*), as well as the formation of heterojunctions through copolymerization (*5*, *8*). Recently, the design of ever more intricate GNR structures has opened new avenues of exploration, including topics such as negative differential resistance (*9*–*12*), spintronics (*13*, *14*), magnetism (*15*–*20*), and quantum information processing (*21*–*23*).

A common ingredient in many new GNR concepts is the controlled formation of low-energy modes and localized states. Methods to generate localized zero-energy modes include the generation of local sublattice imbalances, the linkage of GNR segments having different topological character (*24*–*27*), and the inclusion of heteroatoms in the GNR backbone (*28*–*30*). Recently, the incorporation of nonhexagonal rings into GNRs has also been presented as a method to fabricate new localized states (*31*–*33*). Four-membered rings are an exciting candidate for this type of application due to the strong antiaromatic character of cyclobutadienoid (CBD) groups (*34*–*37*). Nanoribbons with four-membered rings, however, have so far remained elusive due to challenges in their chemical synthesis (*38*). Although [2 + 2] cycloaddition has been shown to generate four-membered rings via on-surface synthesis, it has so far only been used to couple smaller acenes and triphenylenes (*31*, *39*–*42*). The CBD units in these systems exhibit relatively little antiaromaticity due to their fusion pattern (i.e., because the CBD bonds have more single bond character in the dominant resonance structures that maximize the number of Clar sextets), resulting in a large bandgap (*31*, *36*, *43*). An exception is the formation of pyrene-type GNRs from a tetrabromopyrene precursor (*38*), but growth of this GNR was severely hampered by bromine atoms poisoning the catalytic activity of the gold surface and so only very short GNRs could be produced after actively removing halogens during synthesis through hydrogen dosing. Moreover, electronic characterization was not performed, and so the effect of CBD incorporation in those GNRs is so far unknown. More recently, a dehydrofluorination of fluoropolyphenylenes was used to make biphenylene networks exhibiting four-, six-, and eight-membered rings featuring a width-dependent electronic structure (*44*).

Here, we describe a strategy for fabricating linearly fused coronene-CBD GNRs (cor_4_GNR) through surface density–assisted coupling of tetrabromocoronene precursors. This GNR contains highly antiaromatic four-membered rings that greatly affect its electronic properties. We have performed simulations of the cor_4_GNR electronic structure based on density functional theory (DFT) that show how antiaromaticity of the CBD units translates into emergent low-energy electronic bands that would otherwise be absent in fully aromatic nanoribbons. Other characteristic properties include an indirect bandgap and dispersive band states composed of linear chains of p*_x_*- and p*_y_*-type pseudo-atomic orbitals localized on the CBDs. Synthesis of our cor_4_GNRs is implemented through direct contact transfer (DCT) of brominated coronene derivatives followed by thermally activated [2 + 2] cycloaddition. Coupling occurs exclusively in ultradense surface regions, showing that cycloaddition of these molecules is possible without the need for dosed hydrogen in the presence of high surface coverage. The structure of the resulting GNRs was determined by bond-resolved scanning tunneling microscopy (BRSTM), which verified the expected geometry incorporating embedded CBDs. Electronic characterization of the GNRs was performed by scanning tunneling spectroscopy (STS) measurements as well as two-terminal transport through cor_4_GNRs lifted with the tip of the STM. These measurements reveal a 600-meV bandgap with valence band (VB) and conduction band (CB) onsets characterized by states localized predominantly on the CBDs. The wave function patterns revealed by differential conductance mapping are similar to those predicted by DFT calculations and confirm the picture of CBDs forming a linear chain of emergent p*_x_*- and p*_y_*-type pseudo-atomic orbital bands at energies close to the Fermi level.

## RESULTS

### Theoretical analysis of cor_4_GNRs

**Fig. 1.**
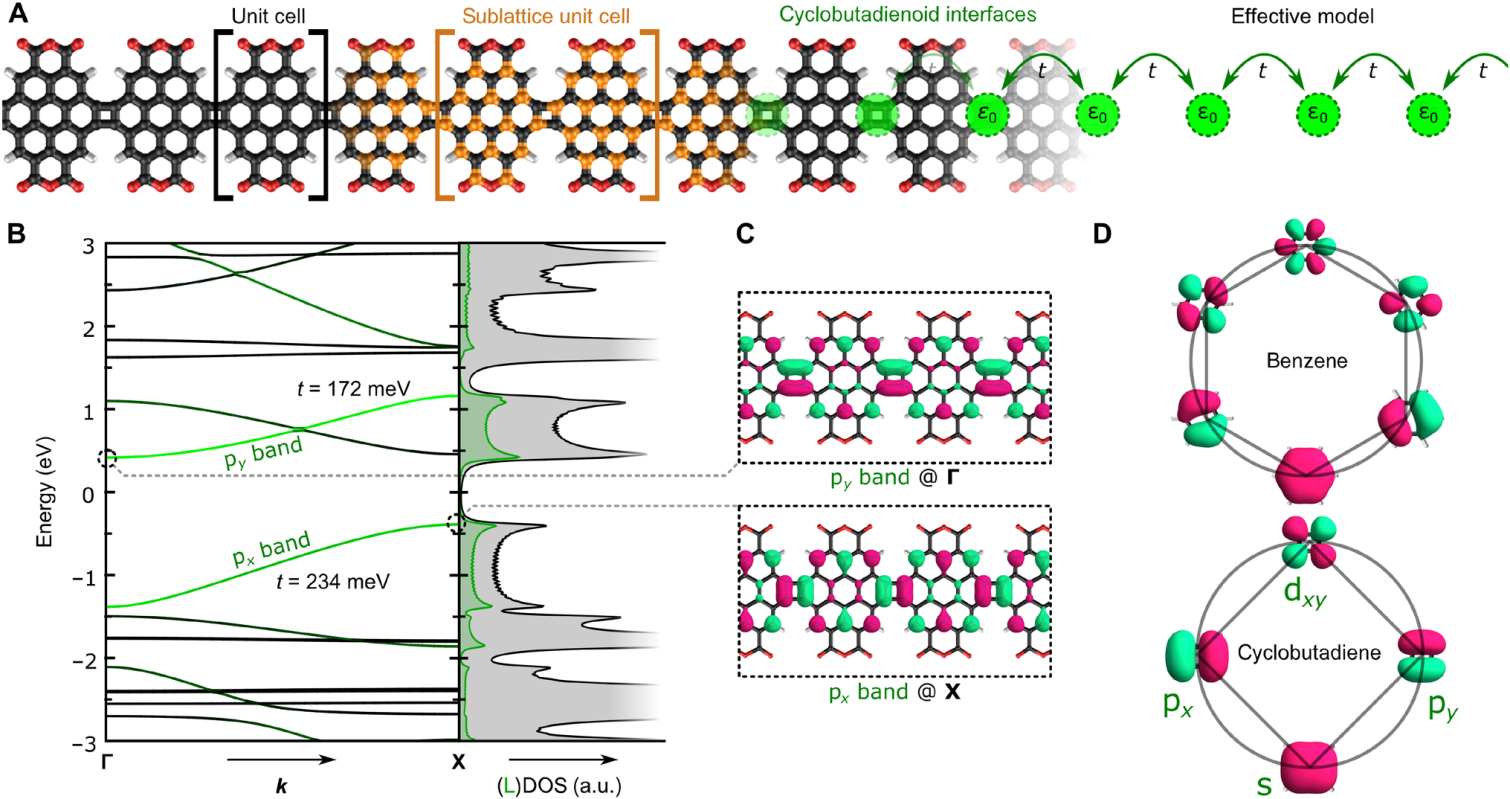
Theoretical analysis of cor_4_GNRs. (**A**) Structure of anhydride-decorated cor_4_GNR with unit cell shown on left. Carbon atoms belonging to the A and B sublattices are shown in black and orange, respectively, while CBD interfaces are highlighted by the green circles. An effective tight-binding model describing the periodic chain of CBD sites is shown on the right. (**B**) Electronic band dispersion and (local) density of states (DOS) of cor_4_GNR in (A). Green shading of the bands indicates the density of the wave function on the carbon atoms of the CBDs. Green (gray) shading in the DOS refers to the local (total) DOS, with the local DOS projected onto the CBDs in arbitrary units (a.u). (**C**) Wave functions for band-edge states plotted at **Γ** and **X**. (**D**) Frost circle representation of π-type frontier states for benzene (top) and cyclobutadiene (bottom).

We start by calculating the DFT electronic structure of a cor_4_GNR containing pendant anhydride groups (**1b**) fused through CBDs as shown in Fig. 1A. The DFT-calculated electronic band dispersion is displayed in Fig. 1B, where the projection of the wave function onto the CBDs is indicated by the green shading of the bands. The ribbon exhibits a pair of bands at low energy with predominant localization on the CBDs. This is also evident in the total (gray) and local (green) densities of states (shown to the right), which reveal band onsets around *E* = ±0.4 eV with high orbital density on the CBDs (resulting in an energy gap of *E*_g_ ≈ 0.8 eV). The GNR orbitals shown in Fig. 1C support the picture of wave function localization at the CBD interfaces and reveal an additional notable feature: the CB states have a single nodal plane on the CBDs aligned with the GNR axis (the *x* direction), whereas the VB states have a nodal plane aligned perpendicular to it (the *y* direction). The orbital patterns on the CBDs are thus reminiscent of p*_y_*- and p*_x_*-type atomic orbitals with an out-of-plane angular momentum of *l* = 1. We therefore refer to them as p*_x_* and p*_y_* pseudo-atomic orbitals.

The emergence of electronic bands relatively close to the Fermi energy (especially considering the narrow size of these GNRs) is a consequence of the antiaromatic nature of the CBDs. This can be understood by considering the π-based frontier orbitals of the most fundamental molecular motifs from which the GNR is composed: hexagonal benzene and tetragonal cyclobutadiene. Figure 1D shows the results of DFT calculations of the orbitals of benzene and cyclobutadiene organized in constructs known as Frost circles, where energy increases along the vertical axis and the horizontal axis is associated with electron angular momentum (*45*). By counting the π-electrons, it can be seen that benzene is gapped, stable, and therefore aromatic at charge neutrality, whereas cyclobutadiene features a degenerate ground state and is thus antiaromatic: a result also known as Hückel’s rule (*46*). Because of its fourfold symmetry, cyclobutadiene can be interpreted as a pseudo-atom with frontier states that are p*_x_* and p*_y_* pseudo-atomic orbitals with an out-of-plane angular momentum of *l* = 1 (Fig. 1D) (*47*). The bonding and antibonding states can similarly be interpreted as s- and d*_xy_*-type orbitals. In regard to the GNR electronic structure, the p*_x_* and p_y_ pseudo-atomic orbitals constitute a basis that dominates the low-energy band structure in an energetic region where the coronene subunits are themselves gapped. In the case of the cor_4_GNR, one black band can be seen to intersect the p*_y_* band, but its wave function is predominantly localized on the anhydride side groups, meaning that the presence of this band is specific to this ribbon and not a general feature of CBD GNRs. Because the CBDs are organized in a linear chain (Fig. 1A), the p*_x_* and p*_y_* pseudo-atomic orbitals hybridize to give a typical linear-chain dispersion of the form *E* = ε_0_ − 2*t* cos(*ka*), where *t* is the effective hopping strength between p*_x_* and p*_y_* basis states. Fitting this tight-binding model to the DFT-calculated p*_x_* and p*_y_* bands of Fig. 1 results in an inter-cyclobutadiene hopping parameter *t* of roughly 200 meV.

Another interesting feature seen in the cor_4_GNR band structure is electron-hole “antisymmetry.” For every bonding state at *E*_F_ − *E* at momentum *k*, there is, to good approximation, an antibonding state at *E*_F_ + *E* at momentum **X** − *k*. Electron-hole symmetry is known to arise naturally from the chiral symmetry of graphene: Every bonding wave function has an antibonding partner at opposite energy relative to the Fermi level. However, the bipartite quality of cor_4_GNRs requires a “sublattice unit cell” that is twice as large as the normal unit cell (Fig. 1A) because the four-membered rings swap the A and B sublattices on adjacent units. Hence, proper electron-hole symmetry in cor_4_GNRs requires the band structure to be folded. This explains why the unfolded band structure (Fig. 1A) is antisymmetric in electron momentum. Electron-hole “antisymmetry” thus dictates that CBD GNRs will be indirect-gap semiconductors.

The emergence of p*_x_* and p*_y_* pseudo-atomic orbital GNR bands with indirect bandgaps is a general property of linked CBDs and is mostly independent of the specific molecular structure that lies between the four-membered rings. This is supported by additional DFT calculations that we performed on GNRs where the anhydride pendant groups were omitted, as well as GNRs where the coronene units were exchanged for pyrene and ovalene units, as shown in fig. S1. In each case, antisymmetric p*_x_* and p*_y_* bands emerge with effective dispersions of the form *E* = ε_0_ − 2*t* cos(*ka*). The incorporation of CBD units into GNRs can thus be viewed as a general strategy for engineering GNRs that have a narrow indirect bandgap and that feature pairs of dispersive bands at low energy. The polyaromatic hydrocarbon spacer elements between the four-membered rings serve only to modulate the bandgap and bandwidth of the emergent p*_x_* and p*_y_* pseudo-atomic bands.

### Synthesis of cor_4_GNRs

**Fig. 2.**
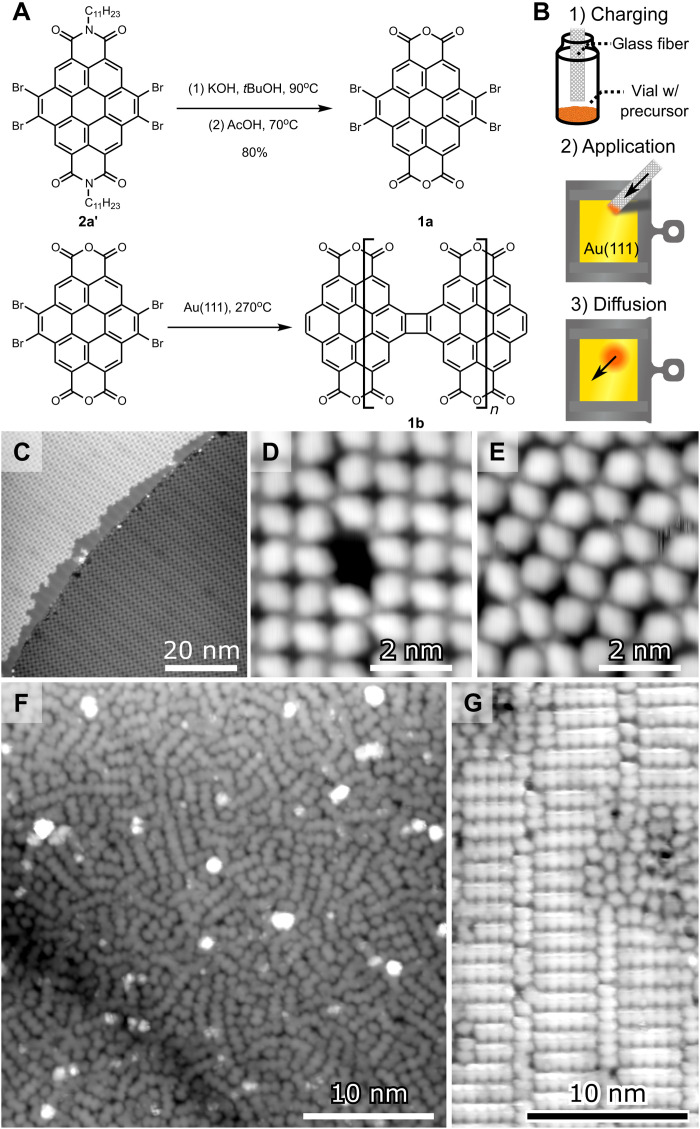
Synthesis of cor_4_GNRs. (**A**) Chemical synthesis of precursor molecule **1a** from alkylated diimide precursor **2a′** (top) and on-surface synthesis of anhydride-functionalized cor_4_GNR **1b** (bottom). (**B**) DCT methodology. (**C**) Large-scale STM topograph of **1a** in type I self-assembly (*V* = −1.8 V and *I* = 50 pA). (**D**) Close-up STM topograph of **1a** in type I self-assembly (*V* = −0.5 V and *I* = 1.5 nA). (**E**) STM topograph of **1a** in type II self-assembly (*V* = −0.5 V and *I* = 1.5 nA). (**F**) Large-scale STM topograph after heating **1a** to *T* = 270°C shows GNR formation (*V* = −1.8 V, *I* = 50 pA). (**G**) Close-up topograph after heating **1a** to *T* = 270°C shows well-ordered GNRs (**1b**) (*V* = −2 V and *I* = 50 pA). STM data obtained at *T* = 4.5 K.

We now discuss the synthesis of cor_4_GNRs, as shown schematically in Fig. 2A. The molecular precursor for the anhydride cor_4_GNR (**1b**) is tetrabromocoronene dianhydride **1a** and was synthesized by saponification of alkylated coronene diimide **2a′** with potassium hydroxide, followed by dehydration in acetic acid [fig. S7; matrix-assisted laser desorption/ionization–time-of-flight (MALDI-TOF) data shown in fig. S8]. The prime in **2a′** denotes the alkylated molecule, to contrast it with the pure tetrabromocoronene diimide **2a**, which was also used for experiments (synthesis and characterization of which are shown in figs. S9 and S10, respectively). We initially attempted to deposit precursor **1a** onto Au(111) through sublimation from a Knudsen cell, but the precursor was unstable and pyrolyzed at elevated temperature before it could sublimate. We therefore turned to DCT to transfer precursor **1a** onto the Au(111) surface, as shown schematically in Fig. 2B (where an applicator directly deposits precursor powder onto the surface via mechanical contact) (*48*). The DCT methodology leads to highly nonhomogeneous samples with molecular coverage ranging from the submonolayer regime to the multilayer regime depending on the distance from the point where the applicator contacts the surface. Gentle heating of the sample to *T* = 200°C caused diffusion of the deposited molecules and resulted in large regions of the surface exhibiting full monolayer (ML) coverage.

Areas having 0.6 to 1.0 ML coverage exhibit rich self-assembly behavior as seen in Fig. 2 (C to E). The dominant van der Waals–bonded structure (type I) is shown in Fig. 2 (C and D) and exhibits fourfold rotational symmetry (*C*_4v_) while remaining chiral because the molecular axes of the oblate precursor **1a** are slanted off the axes of the two-dimensional (2D) crystal lattice [see fig. S2, which shows experimental details for the very similar coronene diimide (**2a**) for more structural details]. Another common morphology (type II) is shown in Fig. 2E and features compression of the molecules in the rows (compared to Fig. 2D) and separation of the molecules in the columns. Other less common self-assembly patterns are shown in fig. S3. Further heating of the sample to *T* = 270°C causes a marked change in morphology as seen in Fig. 2F. Here, the molecules have covalently bonded into ribbon-like structures with 2 to 12 repeating units. Other areas show highly ordered nanoribbon arrays, as shown in Fig. 2G. Conversion of the van der Waals–bonded self-assemblies of Fig. 2 (C to E) into the covalently bonded GNRs of Fig. 2 (F and G) was found almost exclusively in ultradense surface regions, while only sporadic GNR formation was found in areas with coverage between 0.9 and 1 ML (see fig. S4). Growth did not take place at all in areas with coverage below 0.9 ML, with these areas persistently featuring the van der Waals–bonded self-assemblies described above, despite the higher temperature anneal. In some very dense areas, covalently bonded GNRs “crowded” on top of each other, effectively creating GNR-intercalated GNRs (fig. S4). Upon heating further up to *T* > 330°C, some coupling between monomers is observed in the lower-coverage areas, but the coupling becomes nondirectional and results in short, poor-quality graphitic structures, as shown in fig. S3. High surface density appears to play a critical role in the formation of covalently bonded cor_4_GNRs.

### Structural analysis

**Fig. 3.**
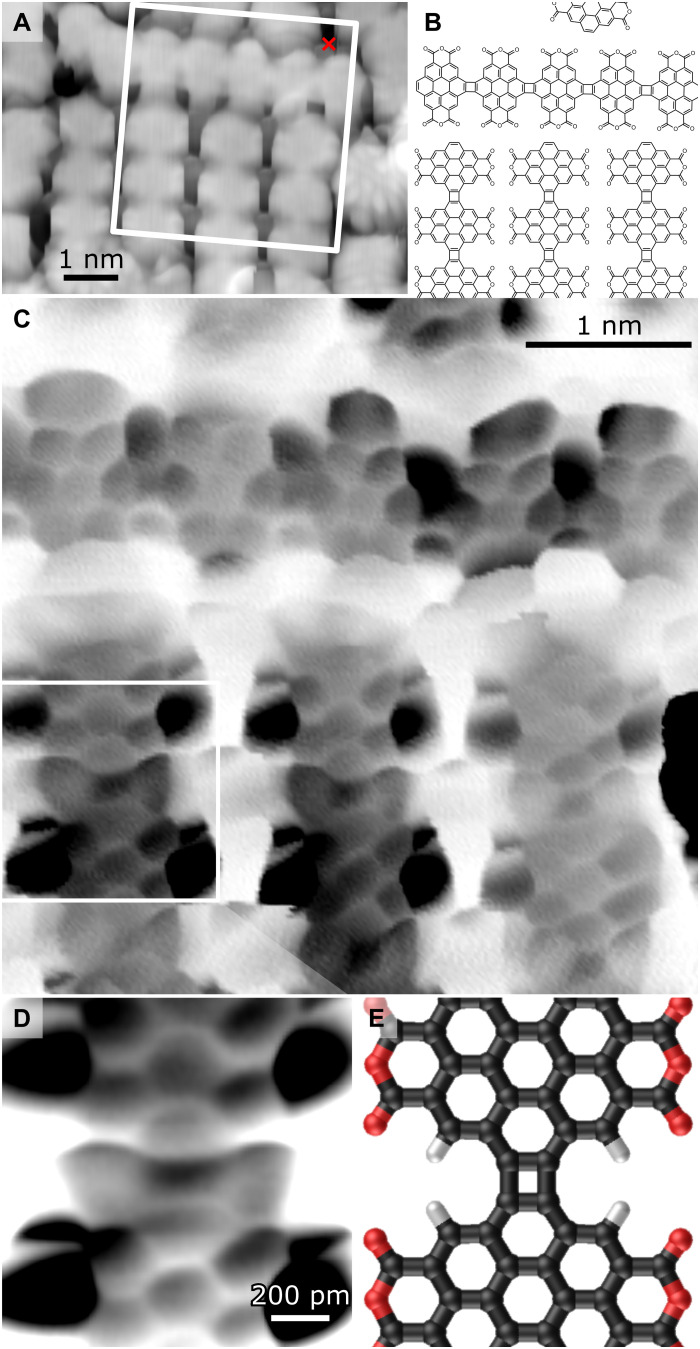
Structural characterization of cor_4_GNRs. (**A**) STM topograph of cor_4_GNRs (*V* = −1 V and *I* = 100 pA). The red cross indicates the position where the tip was attached to the GNR in the lifting experiment described in the “Electronic characterization” section. (**B**) Schematic model of cor_4_GNRs in the region indicated in (A). (**C**) BRSTM scan of the region indicated in (A) (constant height image of the tunneling current; *V* = 50 mV). (**D**) Close-up BRSTM scan of boxed region in (C) shows a CBD ring (constant height image of the tunneling current; *V* = 50 mV). (**E**) Structural model of the GNR image in (D). STM data obtained at *T* = 4.5 K.

To verify that the GNR product from precursor **1a** is the desired [2 + 2] cycloaddition product and not an unwanted fusion product, we performed BRSTM measurements with a molecule-passivated tip (*49*). An STM topograph of a region with cor_4_GNRs is shown in Fig. 3A, and a proposed structural model of the GNRs is shown in Fig. 3B. Figure 3C shows a BRSTM scan of the boxed region of Fig. 3A, which reproduces the carbon-carbon bonds of the proposed model. A close-up scan of a CBD linkage is shown in Fig. 3D, and the corresponding structural model can be seen in Figure 3E. The four-membered ring can be recognized in the center, although the bonds on the side are not well resolved due to a bowtie-like effect in the contrast. This effect is a consequence of the energetic proximity of band states near the Fermi level. The distorted six-membered rings seen at the sides of the GNR are similar to what has been observed before on related anhydride-containing molecules (*50*), although the large contrast seen here between different groups suggests an inhomogeneous interaction with the underlying gold substrate (*51*). The image distortions are tentatively ascribed to interaction of the molecule-passivated tip with the resulting inhomogeneous charge distribution (possibly involving radical formation). Regardless of the edge intricacies, the backbone of the GNR remains well resolved and the GNR can be assigned as a [2 + 2] coupling product with CBDs.

### Electronic characterization

**Fig. 4.**
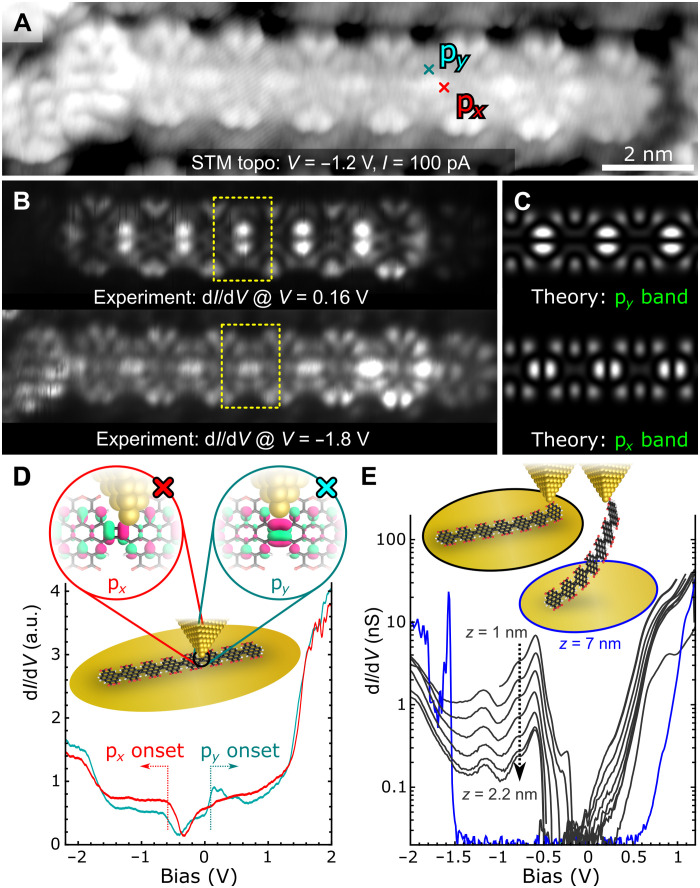
Electronic characterization of cor_4_GNRs. (**A**) STM topograph of an octameric cor_4_GNR (*V* = −1.2 V and *I* = 100 pA). (**B**) Experimental LDOS maps of GNR shown in (A) obtained at *V* = 0.16 V (top) and *V* = −1.8 V (bottom). (**C**) Simulated LDOS maps. (**D**) STS spectra recorded on the GNR shown in (A). The spectra were recorded with the tip centered on a p*_x_* lobe (red) and a p*_y_* lobe (teal) as indicated in (A) and sketched in inset. (**E**) Height-dependent lifting conductance spectra of the GNR marked in Fig. 3A by a red “x.” Inset shows sketch of the GNR lifting process. STM data obtained at *T* = 4.5 K.

We performed in-depth electronic structure measurements on the cor_4_GNRs using STS. Figure 4A shows an STM image of an octameric cor_4_GNR. The wave function distribution in this GNR was determined by mapping the differential conductance at constant height for both positive and negative bias voltages. The results, shown in Fig. 4B, reveal a chain of vertically aligned pairs of bright spots at the locations of the CBD units in the empty-state image (*V* > 0), and a chain of horizontally aligned bright spots at the locations of the CBD units in the filled-state image (*V* < 0). The empty-state d*I*/d*V* map of the CBD units strongly resembles the p*_y_*-band wave function shown in Fig. 1C, whereas the CBD filled-state map resembles the p*_x_*-band wave function (rotated 90° relative to p*_y_*). To confirm the identity of the experimentally measured GNR states, we calculated the theoretical local density of states (LDOS) of the cor_4_GNR for both the empty-state p*_y_* band and the filled-state p*_x_* band (Fig. 4C). These theoretical LDOS maps match the experimental data in Fig. 4B, providing further evidence that we are experimentally observing the p*_x_* and p*_y_* pseudo-atomic orbitals expected to accompany CBD units in a cor_4_GNR. The slight discrepancy between the experimental and theoretical LDOS patterns, e.g., the two-lobe pattern instead of the three-lobe pattern on the sides of the coronene subunits, is due to simultaneous tunneling into electronic states localized on the pendant anhydride groups: those originating from the black band intersecting the green p*_y_* band in the DFT calculations (see Fig. 1B). Their persistence over a large bias range of both positive and negative bias voltages suggests that these states are strongly hybridized with the gold surface.

The energy dependence of the cor_4_GNR states was experimentally probed using d*I*/d*V* mapping, d*I*/d*V* point spectroscopy, and in situ “lifting spectroscopy” (i.e., two-terminal transport). The filled-state p*_x_*-like LDOS pattern shown in the bottom half of Fig. 4B was seen to persist over the bias range −2.1 V < *V* < −0.6 V, while the empty-state p*_y_*-like LDOS shown in the top half of the figure persisted over the range 0.05 V < *V* < 1.2 V, with d*I*/d*V* maps recorded within the energy gap not returning a p-orbital pattern on the CBDs (see fig. S5). This provides evidence for a relatively narrow GNR energy gap of *E*_g_ ≈ 0.6 eV as well as the presence of dispersive p*_x_* and p*_y_* bands, and agrees reasonably well with the theoretical band structure of Fig. 1B. Further evidence for this type of energy dependence can be seen in the d*I*/d*V* point spectroscopy of Fig. 4D, which shows spectroscopy taken at two different points chosen to separately emphasize p*_x_*-band states (red) and p*_y_*-band states (teal) (the spectroscopy positions are marked in the topograph of Fig. 4A). Both spectra show a reasonably clear VB onset at *V* = −0.6 V, but only the blue spectrum shows a conduction band onset near *V* = 0.1 V. Both spectra show higher energy upturns in d*I*/d*V* (i.e., *V* = −1.8 V and *V* = 1.6 V) that likely mark the onset of higher energy bands.

The results of in situ lifting experiments can be seen in Fig. 4E. This technique is useful for gaining insight into the two-terminal transport properties of GNRs, as well as the intrinsic GNR electronic structure in the absence of a substrate (*10*, *52*). Figure 3A shows the ribbon that was lifted by attaching the tip to the side of the last unit of the GNR (marked by a red cross in the figure). Figure 4E shows the two-point transport differential conductance as a function of voltage for low tip heights in the range 1 nm < *z* < 2.2 nm (gray spectra) and for near detachment at *z* = 7 nm (blue spectrum). As the GNR is lifted, the states in the energy gap region (−0.6 V < *V* < 0.1 V) are swept away, implying that they arise mainly from tunneling into the Au(111) surface (consistent with the STS of Fig. 4D). The most prominent feature that remains is a peak at *V* ≈ −0.6 V, which marks the VB onset. The amplitude of this feature decays as *G* = *G*_0_ e^−βΔ*z*^ with a decay parameter β = 2.7 nm^−1^, signaling off-resonant transport (*10*, *52*, *53*). When the ribbon is pulled away from the surface to a height of 7 nm, the VB fingerprint disappears entirely and the CB conductance also decays notably, leaving onsets at *V* = −1.5 V and *V* = 0.8 V. The off-resonant character of the transport through the p*_x_*- and p*_y_*-type states can be understood from their localized nature. When lifted, the initial particle-in-a-box states inside the p*_x_* and p*_y_* bands no longer constitute ballistic transport channels between tip and surface because the individual CBD units may be separated electrostatically, causing the transport to attain a more hopping-like character. At large tip heights, the only remaining transport channels are formed by fully delocalized states that connect well to both tip and surface: states from the black bands of the electron dispersion of Fig. 1B. The experimentally observed exponential decay (with *z*) of the VB feature thus provides additional evidence that it arises from CBD-derived p*_x_*-band states.

## DISCUSSION

These results can be thought of as “inverting” the normal GNR paradigm, where functionality is designed into precursor molecules that become the unit cells for quasi-1D GNR crystals where intercell linkages are of secondary importance. In cor_4_GNRs, it is the linkages between precursor units that are paramount, while the precursor itself has only a secondary influence on the band structure that emerges from the coupling between linkage groups. Our STS results support this picture and demonstrate that four-membered GNR linkages host frontier states that lead to dispersive bands composed of p*_x_* and p*_y_* pseudoatomic orbitals. Other than providing coupling between CBDs, the precursor structure mainly just determines the dielectric environment of the CBD-derived p*_x_* and p*_y_* bands. Two-terminal transport reveals fingerprints of these CBD band states through the decay of the signal at increased tip height. The narrow energy gap of cor_4_GNRs as well as the bandgap’s indirect nature and the dispersive character of the frontier bands are all consequences of the CBD lattice and are not specific to any coronene backbone or pendant groups.

The cor_4_GNR properties described here should be generalizable to other CBD GNRs, as corroborated by our DFT calculations on pyrene- and ovalene-type GNRs (fig. S1). The pseudo-atomic orbital physics of such GNRs could potentially be exploited in new designer quantum systems, an example of which might be the placement of alternating molecular segments between CBD units (shown in fig. S6) to experimentally realize new effective Su-Schrieffer-Heeger models (*27*, *47*, *54*–*56*).

## MATERIALS AND METHODS

### Synthesis

**1a** was prepared from **2a′** (see fig. S7) according to the following procedure: To a 20-ml vial was added **2a′** (20 mg, 0.019 mmol), potassium hydroxide (50 mg, 0.89 mmol), *tert*-butyl alcohol (4 ml), and a magnetic stirring bar. The vial was sealed with a Teflon cap and stirred at *T* = 90°C for 4 hours. Acetic acid (7 ml) was then added to the reaction mixture, and the mixture was stirred at *T* = 70°C for 2 hours. The solid was then filtered and washed with H_2_O, dichloromethane, and acetone to get **1a** as an orange solid (11 mg, 80% yield). Synthesis of **2a** and **2a′**, as well as MALDI-TOF characterization of **1a** and **2a**, is shown in figs. S7 to S10.

### Sample preparation

Atomically clean Au(111) surfaces were prepared through repeated cycles of argon ion (Ar^+^) bombardment and annealing. Tetrabromocoronene dianhydride (**1a**) was introduced onto the surface through DCT using the same setup as described in our previous work (*57*). The glass fiber was cleaned using various solvents and subsequently outgassed in high vacuum (*P* < 10^−7^ mbar) at *T* = 500°C for *t* = 30 min. It was then taken out of the vacuum, and the precursor was applied in powder form. The fiber was then reintroduced into the vacuum and gently heated to a temperature of roughly *T* = 100°C for *t* = 30 min to remove trace solvent impurities and atmospheric contaminants. The precursor was introduced onto the surface by gently stamping the cooled fiber onto it, until a barely visible amount of material was observed by eye.

### STM measurements

All STM experiments were carried out using a commercial Createc LT-STM held at *T* = 4.5 K using platinum-iridium tips. Image processing of the STM scans was performed using WSxM software (*58*). STS and differential conductance mapping experiments were performed with the use of a lock-in amplifier using a wiggle voltage amplitude (*V*_ac_) of 4 to 20 mV at a frequency (*f*) of 620 Hz or 577.7 Hz. Transport experiments were performed according to the procedure described in (*10*). BRSTM measurements were performed by passivating the tip with a molecule from the surface, followed by imaging the current at constant height and low bias. Differential conductance mapping was also performed using a passivated tip. In both cases, the atomic or molecular species passivating the tip is unknown and difficult to control as the full coverage of the samples prohibits the controlled pickup of any identifiable molecule. Passivation was therefore achieved at random by poking the tip around the edges of the GNRs.

### Calculations

Periodic DFT calculations were carried out using the Quantum ESPRESSO package (*59*). All structures were fully relaxed until all force components were smaller than 0.01 eV/Å. We used 60 Ry as the plane-wave cutoff and Perdew-Burke-Ernzerhof (PBE) norm-conserving pseudopotentials. A vacuum region of 15 Å was added in the nonperiodic direction to prevent interactions between replicas. Finite DFT calculations (Fig. 1E) were performed using ORCA (*60*). The geometry was optimized until all force components were smaller than 2.6 10^−4^ eV/Å. The def2-SV(P) basis set was selected together with the B3LYP exchange-correlation functional. Molecular and crystal orbitals were rendered using MathemaTB (*61*).

## Supplementary Materials

### This PDF file includes:

Figs. S1 to S10 Synthetic Details References
