# Supplementary material for: Pseudo-atomic orbital behavior in graphene nanoribbons with four-membered rings
Source: Sci Adv. 2021 Dec 22;7(52):eabl5892. doi: 10.1126/sciadv.abl5892 (PMC8694588; doi:10.1126/sciadv.abl5892)
Supplement: Supplementary file 1 — Figs. S1 to S10 Synthetic Details References [file sciadv.abl5892_sm.pdf]

Science Advances
AAAS

Supplementary Materials for

Pseudo-atomic orbital behavior in graphene nanoribbons with four-membered rings

Peter H. Jacobse*, Zexin Jin, Jingwei Jiang, Samuel Peurifoy, Ziqin Yue, Ziyi Wang, Daniel J. Rizzo, Steven G. Louie*, Colin Nuckolls*, Michael F. Crommie*

*Corresponding author. Email: jacobse@berkeley.edu (P.H.J.); sglouie@berkeley.edu (S.G.L.); cn37@columbia.edu (C.N.); crommie@berkeley.edu (M.F.C.)

Published 22 December 2021, *Sci. Adv.* **7**, eabl5892 (2021)
DOI: 10.1126/sciadv.abl5892

This PDF file includes:

Figs. S1 to S10
Synthetic Details
References

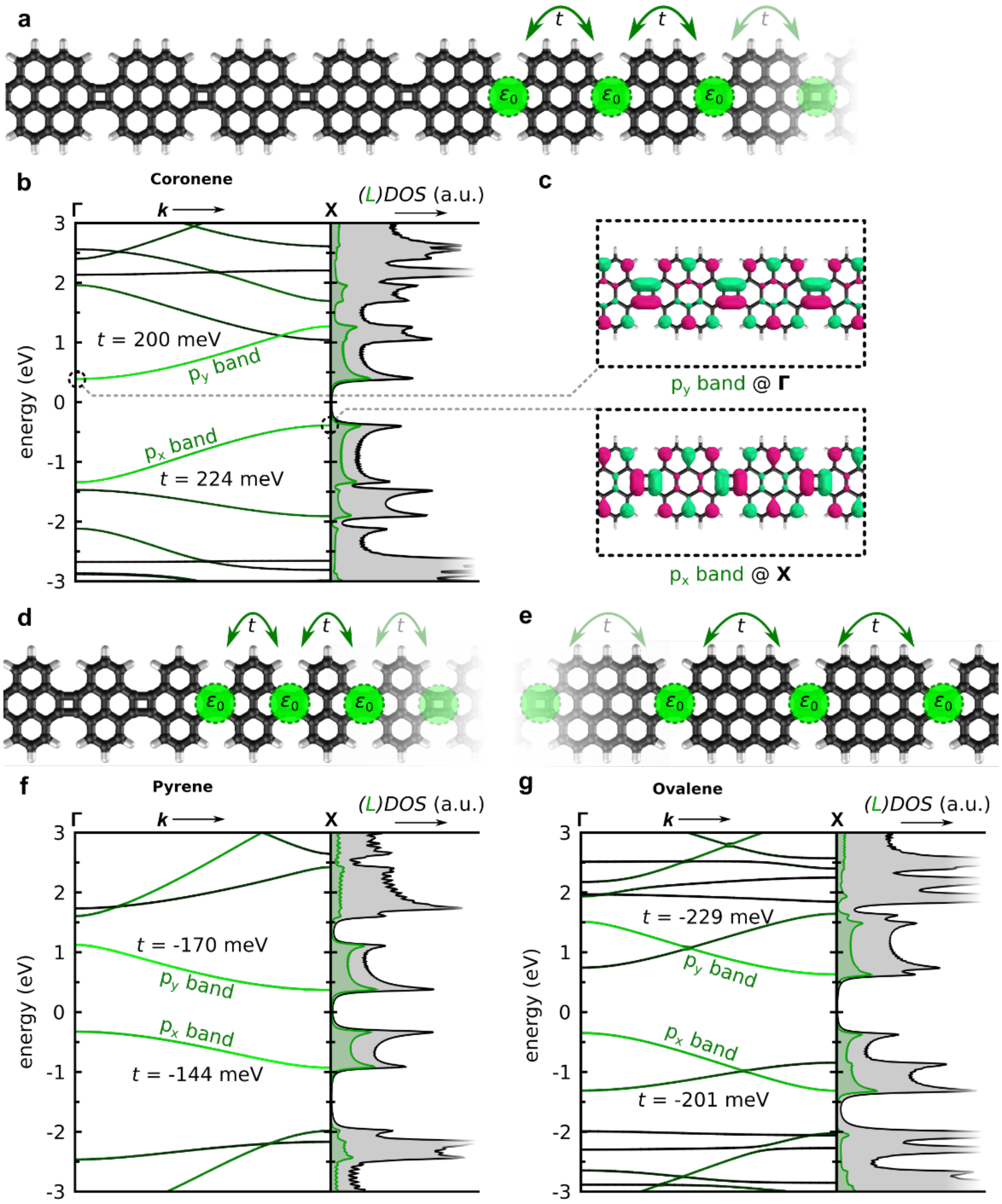

Fig. S1. Electronic structure of various cyclobutanoid GNRs derived from cor4GNR. (a) Structure of cor4GNR without pendant anhydride groups. (b) Electronic band dispersion (left) and density of states (right) of cor4GNR. Hopping parameters derived for the effective tight-binding model are shown. Green shading indicates wave function projection onto the CBDs. (c) Wave functions of the band-edge states. (d) Structure of pyrene cyclobutadienoid GNR (pyr4GNR). (e) Structure of ovalene cyclobutadienoid GNR (ova4GNR). (f) Electronic band dispersion (left) and density of states (right) of pyr4GNR. Hopping parameters for the effective tight-binding model are shown. The green shading indicates wave function projection onto the CBDs. (g) Electronic band dispersion (left) and density of states (right) of ova4GNR. Hopping parameters for the effective tight-binding model are shown. The green shading indicates wave function projection onto the CBDs.

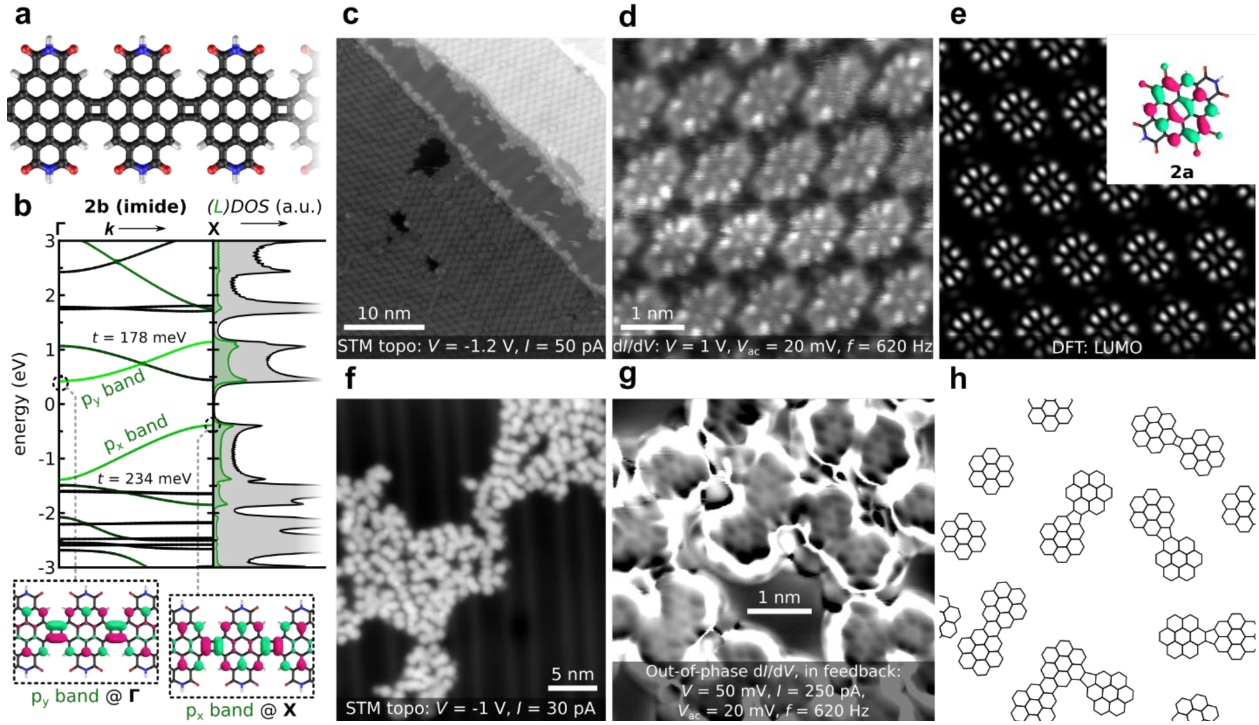

Fig. S2. Electronic structure and attempted synthesis of imide-functionalized cor$_4$GNRs. (a) Target structure of imide-functionalized cor$_4$GNR **2b**. (b) Electronic band dispersion (left) and density of states (right) of **2b**. The green shading indicates wave function projection onto the CBDs. The insets at the bottom show the band-edge wave functions. (c) STM topograph of **2a** after deposition onto Au(111) through sublimation reveals a close-packed structure. (d) Differential conductance map of the self-assembled molecules of **2**a for empty states. (e) Simulated lowest unoccupied molecular orbital (LUMO) of **2a** (top right) and theoretical LDOS for molecular assembly. (f) Result of slow heating (t_{ramp} = 1hr) of the sample shown in (c) and (d) to T = 400 ºC. (g) Bond-resolved STM scan of the structures shown in (f). (h) Schematic model of the bonding of the coronene cores deduced from the BRSTM figure in (g). Scan parameters are indicated on images. STM data obtained at T = 4.5K.

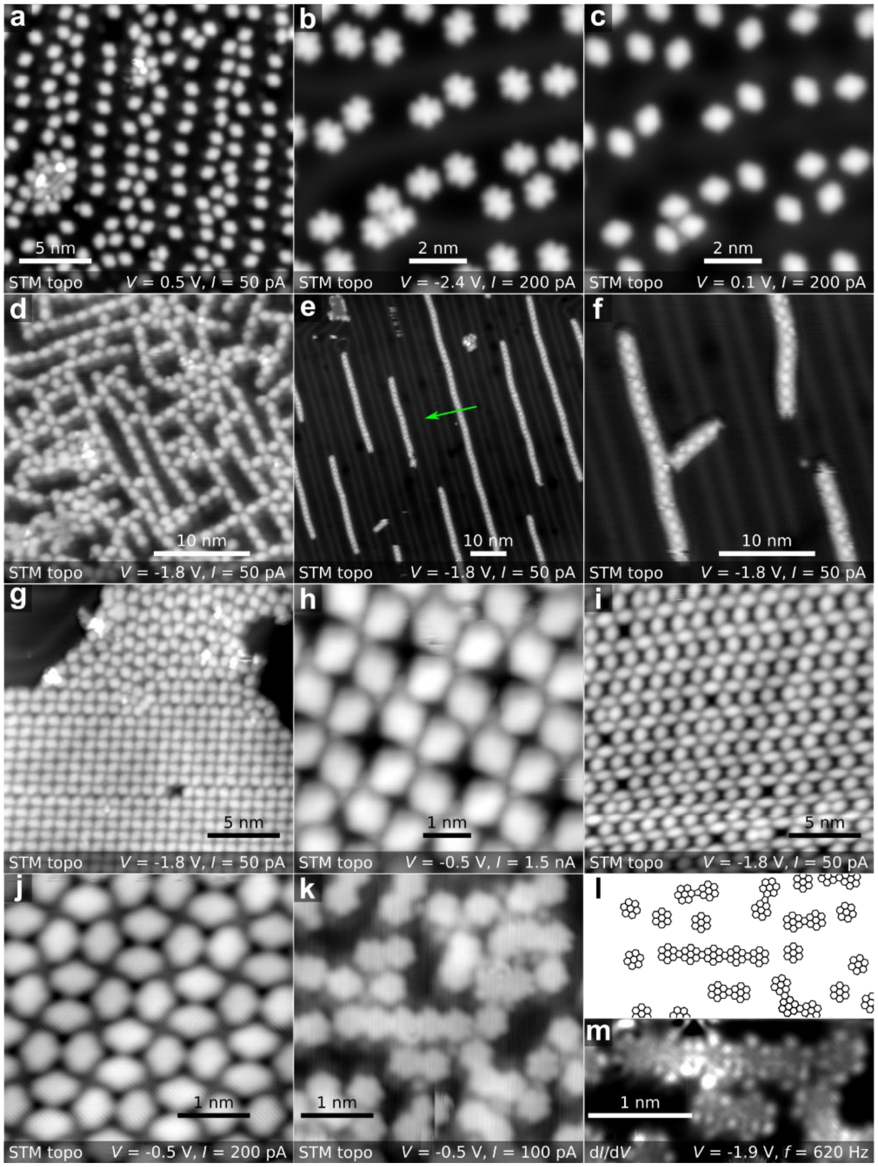

Fig. S3. Self-assembly and on-surface synthesis of anhydride-functionalized precursor 1a. (a) STM topograph of low coverage of **1a** on Au(111). (b) and (c) show close-up STM topographs at different bias voltages. (d) and (e) show chain-like assemblies of **1a** (type III self-assembly). The green arrow in (e) shows tip trajectory for a horizontal tip manipulation experiment to confirm the non-covalent nature of the chain bonding. (f) The result of the tip manipulation experiment in (e) shows an easily broken chain. (g) STM topograph of an area having assemblies of **1a** exhibiting type I self-assembly behavior (bottom) and type IV behavior (top). (h) Close-up STM topograph shows type IV self-assembly of **1a**. (i) STM topograph shows type II self-assembly of **1a**. (j) Close-up STM topograph shows type V self-assembly of **1a**. (k) STM topograph of **1a** in a low-density area after heating to $T = 330$ ºC. (l) Schematic model of coronene core structure based on (k). (m) Differential conductance map of the GNRs in (k) revealing a non-cyclobutadienoid electronic structure, likely due to coupling on the positions that used to connect to the anhydride groups of **1a**. All scan parameters are indicated in the images. STM data obtained at $T = 4.5$K.

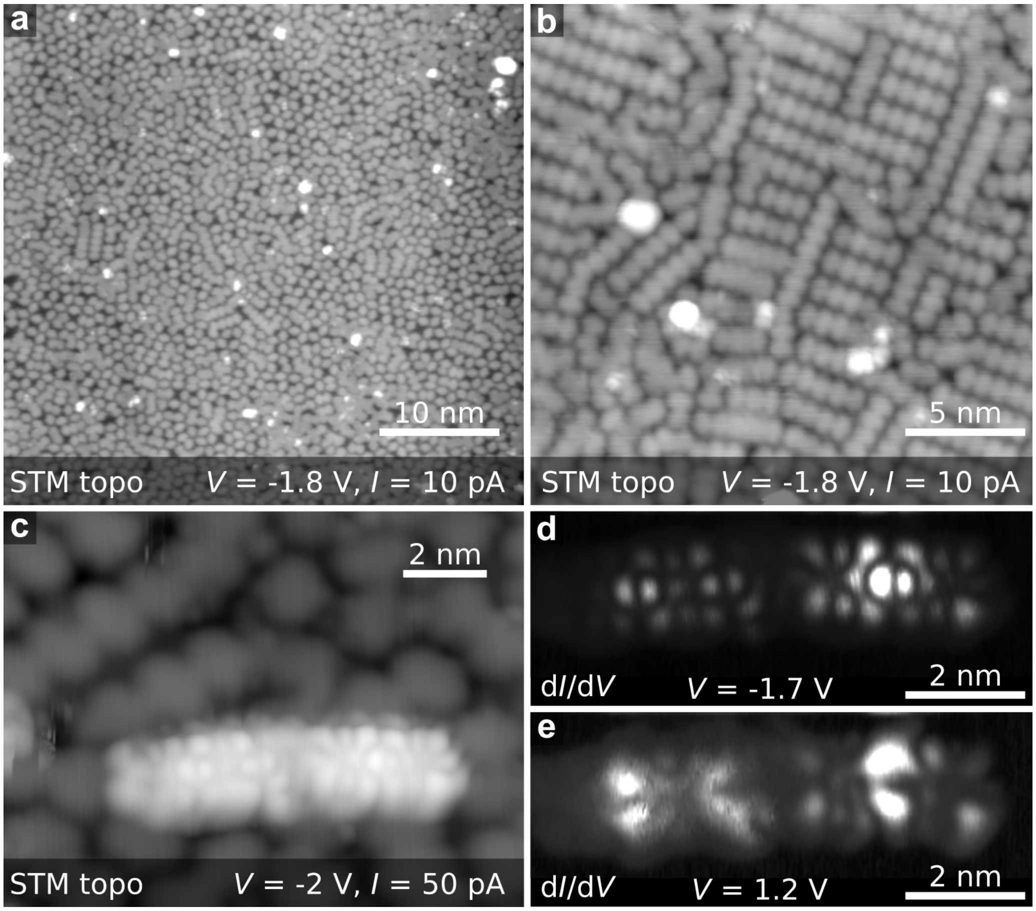

Fig. S4. Effect of surface density on growth of cor4GNRs and emergence of GNR-intercalated cor4GNRs. (a) STM scan of a sample of cor4GNRs prepared through [2 + 2] cycloaddition of anhydride-functionalized precursor **1a**, with a coverage slightly below 1ML. Some GNR growth is observed, but the supermajority of molecules remains monomeric. (b) STM scan of a different area on the same sample with full ML coverage. Here, GNR growth is much more efficient, and longer GNRs are observed while uncoupled monomers are now scarce. The propensity for GNRs to assemble in ordered arrays is also increased. (c) STM scan of an area of the same sample, which is locally dense enough such that nanoribbons are pushed up on top of each other. The hexamer in the scan is partially lying on top of other GNRs, although part of its left side is still touching the Au(111) surface. (d) and (e) show constant height differential conductance maps at two different bias voltages (V = -1.7V, 1.2V, V_{ac} = 10 mV, f = 620 Hz) recorded on the GNR-intercalated cor4GNR in (c). STM data obtained at T = 4.5K.

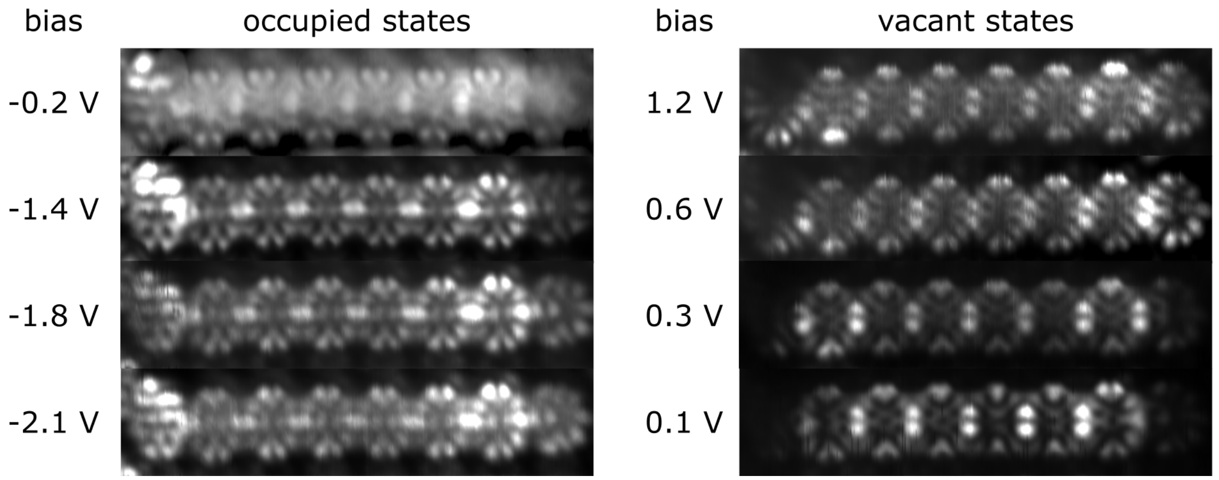

Fig. S5. More experimental LDOS maps on a cor4GNR. The left column shows maps recorded at negative bias voltages (occupied states) while the right column shows maps recorded at positive bias voltage (vacant states), all taken on the cor4GNR of Figure 4 of the main text. Imaging was performed at constant height, using a V_{ac} = 10mV wiggle voltage at f = 577.7Hz. The maps recorded at bias voltages down to V = -2.1V show the characteristic p_x pseudo-atomic orbital pattern, while those above V = 0.1V display the characteristic p_y pseudo-atomic orbital pattern. The map recorded at V = -0.2V shows some enhanced (non-resonant) contrast on the four-membered rings, but no characteristic pattern, suggesting it lies within the p_x-p_y band gap.

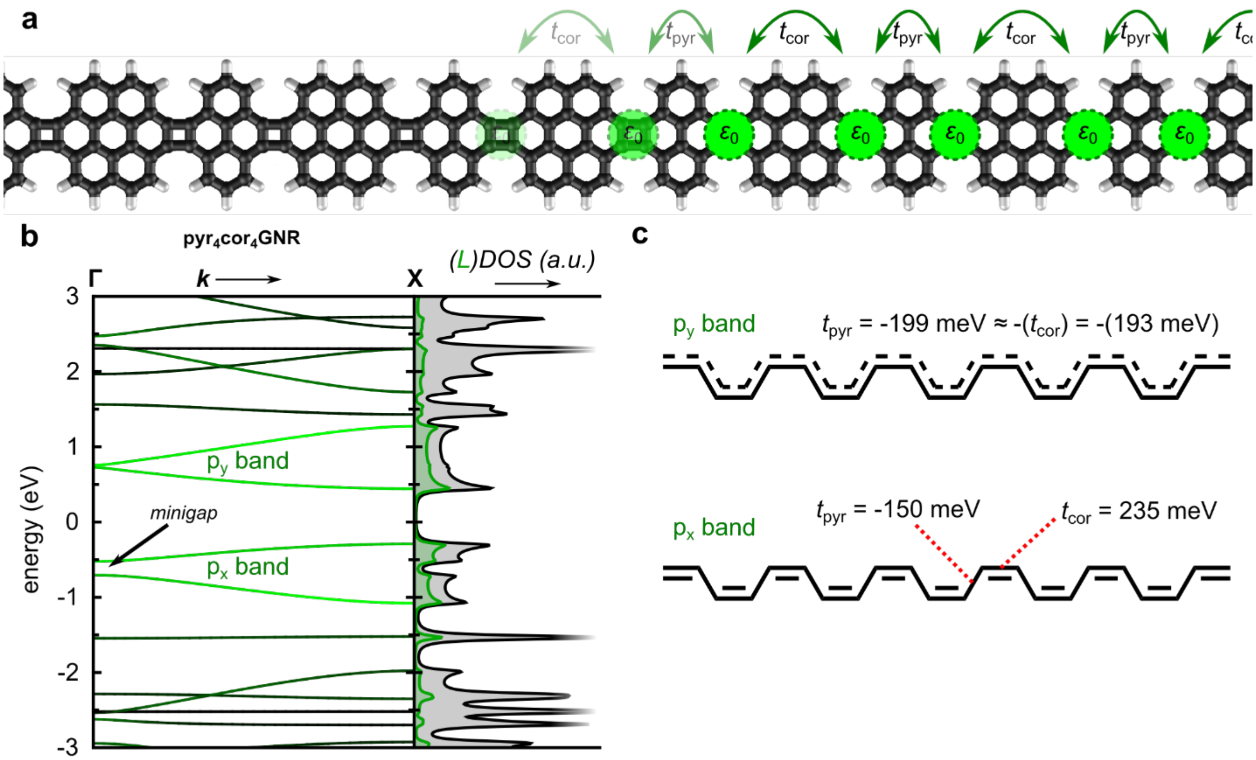

Fig. S6. GNR-intercalated cor4GNRs. Theoretical analysis of proposed SSH chains in a pyrene-coronene-cyclobutadienoid GNR copolymer (pyr4cor4GNR). (a) Structure of a hypothetical, alternating pyr4cor4GNR. (b) Electronic band dispersion (left) and density of states (right) of pyr4cor4GNR. The green shading of the bands indicates wave function projected onto the CBDs. (c) Effective polyacetylene-like model of the p_x and p_y bands (tight-binding parameters for CBD coupling across a pyrene/coronene segment are indicated). The effective two-site dispersion is given by the formula:

$$E = \varepsilon_0 + \sqrt{\left(t_{\mathrm{pyr}}\right)^2 + (t_{\mathrm{cor}})^2 + 2t_{\mathrm{pyr}}t_{\mathrm{cor}}\cos ka}.$$

Synthetic details

Materials. All chemicals were obtained from commercial sources and used as received unless otherwise noted. **2a'** was prepared according to the literature procedure.(*62*)

MALDI-TOF. The mass spectroscopic data were obtained at the Columbia University mass spectrometry facility using a Bruker ultrafleXtreme MALDI TOF/TOF with a frequency-tripled Nd:YAG laser (355 nm).

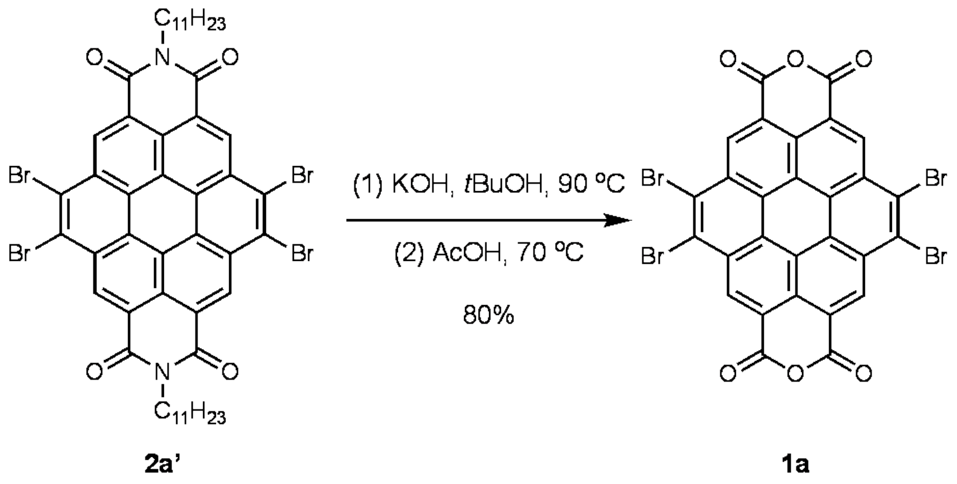

Fig. S7. Synthesis of 1a

*Synthesis of **1a** (5,6,12,13-tetrabromoperyleno[2,1,12-defg:8,7,6-d'e'f'g']diisochromene-1,3,8,10-tetraone).* To a 20 ml vial was added **2a'** (20 mg, 0.019 mmol), potassium hydroxide (50 mg, 0.89 mmol), *tert*-butyl alcohol (4 ml) and a magnetic stirring bar. The vial was then sealed with a Teflon cap and stirred at $T = 90$ºC for 4 hours. Then, acetic acid (7 ml) was added to the reaction mixture and the mixture was stirred at $T = 70$ºC for 2 hours. The solid was then filtered and washed with H_2O, dichloromethane and acetone, to get **1a** as an orange solid (11 mg, 80% yield).

HRMS (MALDI-TOF) calculated m/z for $[C_{28}H_4Br_4O_6]^-$ is 751.6736; found 751.6973.

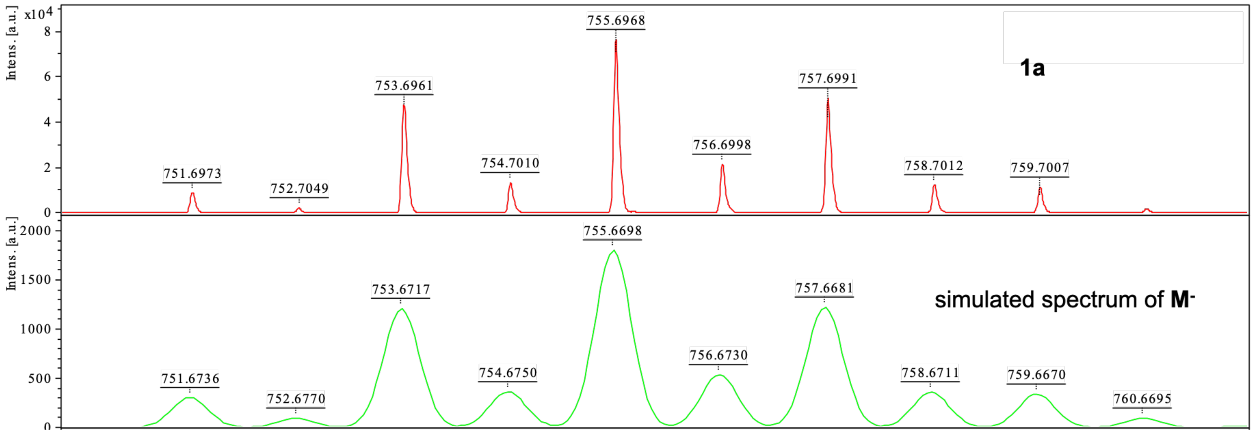

Fig. S8. MALDI-TOF spectrum of 1a.

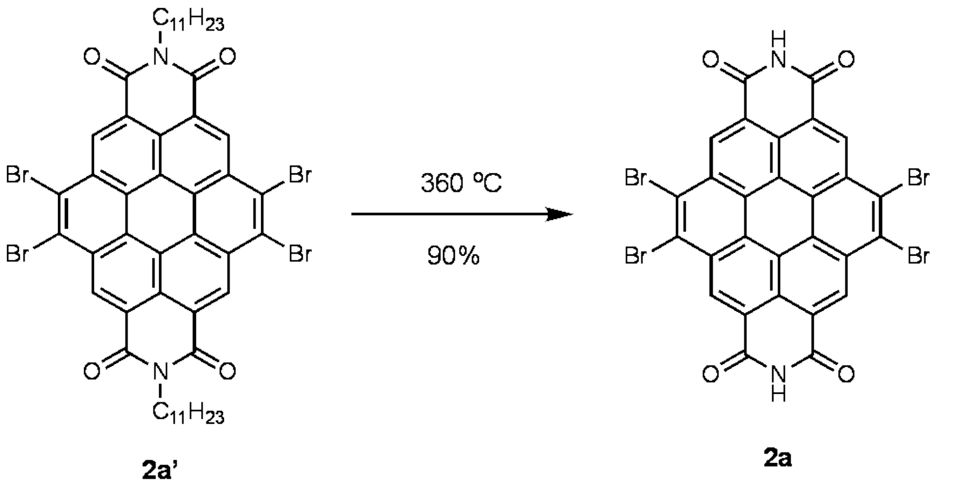

Fig. S9. Synthesis of 2a

Synthesis of ***2a*** *(5,6,12,13-tetrabromoperyleno[2,1,12-defg:8,7,6-d'e'f'g']diisoquinoline-1,3,8,10(2H,9H)-tetraone).* A 4 ml vial charged with **2a'** (15 mg, 0.014 mmol) was sealed in a borosilicate glass tube under vacuum. The tube was placed in a tube furnace, with one end of the tube sticking out of the furnace and the other end containing the solid in the middle of the furnace. The furnace was heated to $T = 360$ºC for $t = 4$ hours, over which time the material turned black and a clear, yellow liquid condensed at the cool end of the tube. The tube was opened and **2a** was collected as a black solid (9.5 mg, 90% yield).

HRMS (MALDI-TOF) calculated m/z for $[C_{28}H_6Br_4N_2O_4]^-$ is 749.7056; found 749.6506.

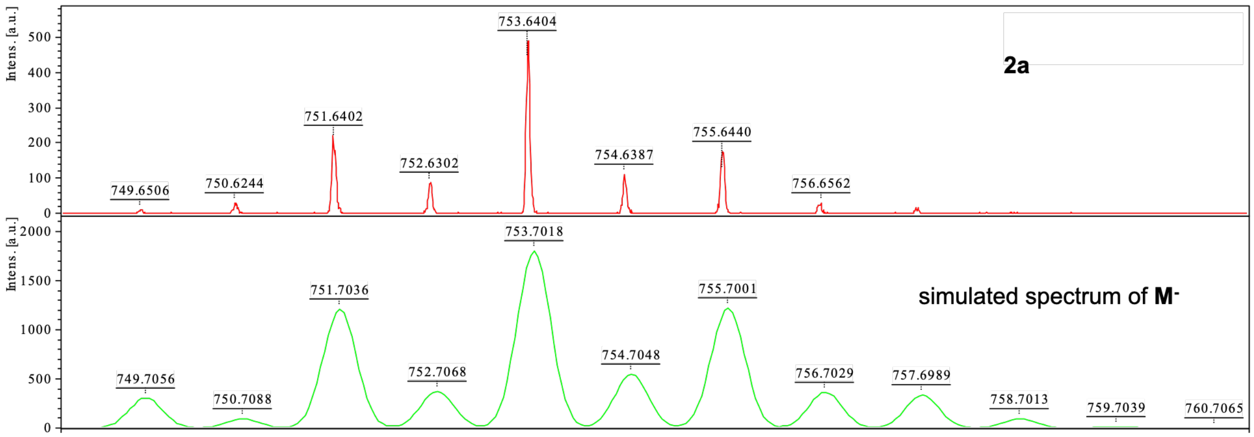

Fig. S10. MALDI-TOF spectrum of 2a.

REFERENCES AND NOTES

1. J. Cai, P. Ruffieux, R. Jaafar, M. Bieri, T. Braun, S. Blankenburg, M. Muoth, A. P. Seitsonen, M. Saleh, X. Feng, K. Müllen, R. Fasel, Atomically precise bottom-up fabrication of graphene nanoribbons. *Nature* **466**, 470–473 (2010).

2. L. Talirz, P. Ruffieux, R. Fasel, On-surface synthesis of atomically precise graphene nanoribbons. *Adv. Mater.* **28**, 6222–6231 (2016).

3. F. Schwierz, Graphene transistors. *Nat. Nanotechnol.* **5**, 487–496 (2010).

4. P. B. Bennett, Z. Pedramrazi, A. Madani, Y.-C. Chen, D. G. de Oteyza, C. Chen, F. R. Fischer, M. F. Crommie, J. Bokor, Bottom-up graphene nanoribbon field-effect transistors. *Appl. Phys. Lett.* **103**, 253114 (2013).

5. C. Bronner, S. Stremlau, M. Gille, F. Brauße, A. Haase, S. Hecht, P. Tegeder, Aligning the band gap of graphene nanoribbons by monomer doping. *Angew. Chemie Int. Ed.* **52**, 4422–4425 (2013).

6. N. Merino-Díez, A. Garcia-Lekue, E. Carbonell-Sanromà, J. Li, M. Corso, L. Colazzo, F. Sedona, D. Sánchez-Portal, J. I. Pascual, D. G. De Oteyza, Width-dependent band gap in armchair graphene nanoribbons reveals fermi level pinning on Au(111). *ACS Nano* **11**, 11661–11668 (2017).

7. R. S. K. Houtsma, J. de la Rie, M. Stöhr, Atomically precise graphene nanoribbons: Interplay of structural and electronic properties. *Chem. Soc. Rev.* **50**, 6541–6568 (2021).

8. J. Cai, C. A. Pignedoli, L. Talirz, P. Ruffieux, H. Söde, L. Liang, V. Meunier, R. Berger, R. Li, X. Feng, K. Müllen, R. Fasel, Graphene nanoribbon heterojunctions. *Nat. Nanotechnol.* **9**, 896–900 (2014).

9. S. Smith, J.-P. Llinás, J. Bokor, S. Salahuddin, Negative differential resistance and steep switching in chevron graphene nanoribbon field-effect transistors. *IEEE Electron Device Lett.* **39**, 143–146 (2018).

10. P. H. Jacobse, A. Kimouche, T. Gebraad, M. M. Ervasti, J. M. Thijssen, P. Liljeroth, I. Swart, Electronic components embedded in a single graphene nanoribbon. *Nat. Commun.* **8**, 119 (2017).

11. D. Nozaki, Lokamani, A. Santana-Bonilla, A. Dianat, R. Gutierrez, G. Cuniberti, Switchable negative differential resistance induced by quantum interference effects in porphyrin-based molecular junctions. *J. Phys. Chem. Lett.* **6**, 3950–3955 (2015).

12. H. Ren, Q.-X. Li, Y. Luo, J. Yang, Graphene nanoribbon as a negative differential resistance device. *Appl. Phys. Lett.* **94**, 173110 (2009).

13. P. Ruffieux, S. Wang, B. Yang, C. Sanchez-Sanchez, J. Liu, T. Dienel, L. Talirz, P. Shinde, C. A. Pignedoli, D. Passerone, T. Dumslaff, X. Feng, K. Müllen, R. Fasel, On-surface synthesis of graphene nanoribbons with zigzag edge topology. *Nature* **531**, 489–492 (2016).

14. X. Kong, B. Cui, W. Zhao, J. Zhao, D. Li, D. Liu, Spin negative differential resistance and high spin filtering behavior realized by devices based on graphene nanoribbons and graphitic carbon nitrides. *Org. Electron.* **15**, 3674–3680 (2014).

15. O. V. Yazyev, Emergence of magnetism in graphene materials and nanostructures. *Rep. Prog. Phys.* **73**, 56501 (2010).

16. S. Mishra, D. Beyer, K. Eimre, S. Kezilebieke, R. Berger, O. Gröning, C. A. Pignedoli, K. Müllen, P. Liljeroth, P. Ruffieux, X. Feng, R. Fasel, Topological frustration induces unconventional magnetism in a nanographene. *Nat. Nanotechnol.* **15**, 22–28 (2020).

17. J. Li, S. Sanz, M. Corso, D. J. Choi, D. Peña, T. Frederiksen, J. I. Pascual, Single spin localization and manipulation in graphene open-shell nanostructures. *Nat. Commun.* **10**, 200 (2019).

18. M. Slota, A. Keerthi, W. K. Myers, E. Tretyakov, M. Baumgarten, A. Ardavan, H. Sadeghi, C. J. Lambert, A. Narita, K. Müllen, L. Bogani, Magnetic edge states and coherent manipulation of graphene nanoribbons. *Nature* **557**, 691–695 (2018).

19. Y. Zheng, C. Li, C. Xu, D. Beyer, X. Yue, Y. Zhao, G. Wang, D. Guan, Y. Li, H. Zheng, C. Liu, J. Liu, X. Wang, W. Luo, X. Feng, S. Wang, J. Jia, Designer spin order in diradical nanographenes. *Nat. Commun.* **11**, 6076 (2020).

20. S. Song, J. Su, M. Telychko, J. Li, G. Li, Y. Li, C. Su, J. Wu, J. Lu, On-surface synthesis of graphene nanostructures with π-magnetism. *Chem. Soc. Rev.* **50**, 3238–3262 (2021).

21. C.-C. Chen, Y.-C. Chang, Theoretical studies of graphene nanoribbon quantum dot qubits. *Phys. Rev. B* **92**, 245406 (2015).

22. Y. Tan, X.-S. Xia, X.-L. Liao, J.-B. Li, H.-H. Zhong, S. Liang, S. Xiao, L.-H. Liu, J.-H. Luo, M.-D. He, L.-Q. Chen, A highly-flexible bistable switch based on a suspended monolayer Z-shaped graphene nanoribbon nanoresonator. *Carbon* **157**, 724–730 (2020).

23. G.-P. Guo, Z.-R. Lin, X.-P. Li, T. Tu, G. Cao, G.-C. Guo, Quantum computation with graphene nanoribbon. *New J. Phys.* **11**, 123005 (2009).

24. T. Cao, F. Zhao, S. G. Louie, Topological phases in graphene nanoribbons: Junction states, spin centers, and quantum spin chains. *Phys. Rev. Lett.* **119**, 076401 (2017).

25. Y. L. Lee, F. Zhao, T. Cao, J. Ihm, S. G. Louie, Topological phases in cove-edged and chevron graphene nanoribbons: Geometric structures, Z 2 invariants, and junction states. *Nano Lett.* **18**, 7247–7253 (2018).

26. D. J. Rizzo, G. Veber, T. Cao, C. Bronner, T. Chen, F. Zhao, H. Rodriguez, S. G. Louie, M. F. Crommie, F. R. Fischer, Topological band engineering of graphene nanoribbons. *Nature* **560**, 204–208 (2018).

27. O. Gröning, S. Wang, X. Yao, C. A. Pignedoli, G. Borin Barin, C. Daniels, A. Cupo, V. Meunier, X. Feng, A. Narita, K. Müllen, P. Ruffieux, R. Fasel, Engineering of robust topological quantum phases in graphene nanoribbons. *Nature* **560**, 209–213 (2018).

28. R. R. Cloke, T. Marangoni, G. D. Nguyen, T. Joshi, D. J. Rizzo, C. Bronner, T. Cao, S. G. Louie, M. F. Crommie, F. R. Fischer, Site-specific substitutional boron doping of semiconducting armchair graphene nanoribbons. *J. Am. Chem. Soc.* **137**, 8872–8875 (2015).

29. S. Kawai, S. Saito, S. Osumi, S. Yamaguchi, A. S. Foster, P. Spijker, E. Meyer, Atomically controlled substitutional boron-doping of graphene nanoribbons. *Nat. Commun.* **6**, 8098 (2015).

30. S. Kawai, S. Nakatsuka, T. Hatakeyama, R. Pawlak, T. Meier, J. Tracey, E. Meyer, A. S. Foster, Multiple heteroatom substitution to graphene nanoribbon. *Sci. Adv.* **4**, eaar7181 (2018).

31. C. Sánchez-Sánchez, T. Dienel, A. Nicolaï, N. Kharche, L. Liang, C. Daniels, V. Meunier, J. Liu, X. Feng, K. Müllen, J. R. Sánchez-Valencia, O. Gröning, P. Ruffieux, R. Fasel, On-surface synthesis and characterization of acene-based nanoribbons incorporating four-membered rings. *Chem. A Eur. J.* **25**, 12074–12082 (2019).

32. M. Liu, M. Liu, L. She, Z. Zha, J. Pan, S. Li, T. Li, Y. He, Z. Cai, J. Wang, Y. Zheng, X. Qiu, D. Zhong, Graphene-like nanoribbons periodically embedded with four- and eight-membered rings. *Nat. Commun.* **8**, 14924 (2017).

33. P. H. Jacobse, R. D. McCurdy, J. Jiang, D. J. Rizzo, G. Veber, P. Butler, R. Zuzak, S. G. Louie, F. R. Fischer, M. F. Crommie, Bottom-up assembly of nanoporous graphene with emergent electronic states. *J. Am. Chem. Soc.* **142**, 13507–13514 (2020).

34. Y. C. Teo, Z. Jin, Y. Xia, Synthesis of cyclobutadienoid-fused phenazines with strongly modulated degrees of antiaromaticity. *Org. Lett.* **20**, 3300–3304 (2018).

35. P. Cui, Q. Zhang, H. Zhu, X. Li, W. Wang, Q. Li, C. Zeng, Z. Zhang, Carbon tetragons as definitive spin switches in narrow zigzag graphene nanoribbons. *Phys. Rev. Lett.* **116**, 26802 (2016).

36. Z. Jin, Y. C. Teo, S. J. Teat, Y. Xia, Regioselective synthesis of [3]naphthylenes and tuning of their antiaromaticity. *J. Am. Chem. Soc.* **139**, 15933–15939 (2017).

37. O. Š. Miljanić, K. P. C. Vollhardt, [N]Phenylenes: A novel class of cyclohexatrienoid hydrocarbon, in *Carbon-Rich Compounds* (Wiley-VCH Verlag GmbH & Co. KGaA, 2006), pp. 140–197.

38. B. V Tran, T. A. Pham, M. Grunst, M. Kivala, M. Stöhr, Surface-confined [2 + 2] cycloaddition towards one-dimensional polymers featuring cyclobutadiene units. *Nanoscale* **9**, 18305–18310 (2017).

39. R. Zhang, B. Xia, H. Xu, N. Lin, Kinetically controlled synthesis of four- and six-member cyclic products via sequential aryl-aryl coupling on a Au(111) surface. *ChemPhysChem* **20**, 2292–2296 (2019).

40. R. Zhang, B. Xia, H. Xu, N. Lin, Identifying multinuclear organometallic intermediates in on-surface [2+2] cycloaddition reactions. *Angew. Chemie Int. Ed.* **58**, 16485–16489 (2019).

41. M. S. G. Mohammed, J. Lawrence, F. García, P. Brandimarte, A. Berdonces-Layunta, D. Pérez, D. Sánchez-Portal, D. Peña, D. G. de Oteyza, From starphenes to non-benzenoid linear conjugated polymers by substrate templating. *Nanoscale Adv.* **3**, 2351–2358 (2021).

42. D.-Y. Li, X. Qiu, S.-W. Li, Y.-T. Ren, Y.-Ch. Zhu, C.-H. Shu, X.-Y. Hou, M. Liu, X.-Q. Shi, X. Qiu, P.-N. Liu, Ladder phenylenes synthesized on Au(111) surface via selective [2+2] cycloaddition. *J. Am. Chem. Soc.* **143**, 12955–12960 (2021)

43. C. K. Frederickson, L. N. Zakharov, M. M. Haley, Modulating paratropicity strength in diareno-fused antiaromatics. *J. Am. Chem. Soc.* **138**, 16827–16838 (2016).

44. Q. Fan, L. Yan, M. W. Tripp, O. Krejčí, S. Dimosthenous, S. R. Kachel, M. Chen, A. S. Foster, U. Koert, P. Liljeroth, J. M. Gottfried, Biphenylene network: A nonbenzenoid carbon allotrope. *Science* **372**, 852–856 (2021)

45. A. A. Frost, B. Musulin, A mnemonic device for molecular orbital energies. *J. Chem. Phys.* **21**, 572–573 (1953).

46. E. Hückel, Quantentheoretische Beiträge zum Benzolproblem. *Z. Phys.* **70**, 204–286 (1931).

47. M. R. Slot, S. N. Kempkes, E. J. Knol, W. M. J. van Weerdenburg, J. J. van den Broeke, D. Wegner, D. Vanmaekelbergh, A. A. Khajetoorians, C. Morais Smith, I. Swart, *p*-band engineering in artificial electronic lattices. *Phys. Rev. X* **9**, 11009 (2019).

48. J. D. Teeter, P. S. Costa, P. Zahl, T. H. Vo, M. Shekhirev, W. Xu, X. C. Zeng, A. Enders, A. Sinitskii, Dense monolayer films of atomically precise graphene nanoribbons on metallic substrates enabled by direct contact transfer of molecular precursors. *Nanoscale* **9**, 18835–18844 (2017).

49. G. D. Nguyen, H. Z. Tsai, A. A. Omrani, T. Marangoni, M. Wu, D. J. Rizzo, G. F. Rodgers, R. R. Cloke, R. A. Durr, Y. Sakai, F. Liou, A. S. Aikawa, J. R. Chelikowsky, S. G. Louie, F. R. Fischer, M. F. Crommie, Atomically precise graphene nanoribbon heterojunctions from a single molecular precursor. *Nat. Nanotechnol.* **12**, 1077–1082 (2017).

50. C. Weiss, C. Wagner, C. Kleimann, M. Rohlfing, F. S. Tautz, R. Temirov, Imaging pauli repulsion in scanning tunneling microscopy. *Phys. Rev. Lett.* **105**, 86103 (2010).

51. P. Hapala, M. Švec, O. Stetsovych, N. J. van der Heijden, M. Ondráček, J. van der Lit, P. Mutombo, I. Swart, P. Jelínek, Mapping the electrostatic force field of single molecules from high-resolution scanning probe images. *Nat. Commun.* **7**, 11560 (2016).

52. M. Koch, F. Ample, C. Joachim, L. Grill, Voltage-dependent conductance of a single graphene nanoribbon. *Nat. Nanotechnol.* **7**, 713–717 (2012).

53. P. H. Jacobse, M. J. J. Mangnus, S. J. M. Zevenhuizen, I. Swart, Mapping the conductance of electronically decoupled graphene nanoribbons. *ACS Nano* **12**, 7048–7056 (2018).

54. M. N. Huda, S. Kezilebieke, P. Liljeroth, Designer flat bands in quasi-one-dimensional atomic lattices. *Phys. Rev. Res.* **2**, 043426 (2020).

55. R. Drost, T. Ojanen, A. Harju, P. Liljeroth, Topological states in engineered atomic lattices. *Nat. Phys.* **13**, 668–671 (2017).

56. L. Yan, P. Liljeroth, Engineered electronic states in atomically precise artificial lattices and graphene nanoribbons. *Adv. Phys. X* **4**, 1651672 (2019).

57. R. D. McCurdy, P. H. Jacobse, I. Piskun, G. C. Veber, D. J. Rizzo, R. Zuzak, Z. Mutlu, J. Bokor, M. F. Crommie, F. R. Fischer, Synergetic bottom-up synthesis of graphene nanoribbons by matrix-assisted direct transfer. *J. Am. Chem. Soc.* **143**, 4174–4178 (2021).

58. I. Horcas, R. Fernández, J. M. Gómez-Rodríguez, J. Colchero, J. Gómez-Herrero, A. M. Baro, WSXM: A software for scanning probe microscopy and a tool for nanotechnology. *Rev. Sci. Instrum.* **78**, 013705 (2007).

59. P. Giannozzi, S. Baroni, N. Bonini, M. Calandra, R. Car, C. Cavazzoni, D. Ceresoli, G. L. Chiarotti, M. Cococcioni, I. Dabo, A. Dal Corso, S. de Gironcoli, S. Fabris, G. Fratesi, R. Gebauer, U. Gerstmann, C. Gougoussis, A. Kokalj, M. Lazzeri, L. Martin-Samos, N. Marzari, F. Mauri, R. Mazzarello, S. Paolini, A. Pasquarello, L. Paulatto, C. Sbraccia, S. Scandolo, G. Sclauzero, A. P. Seitsonen, A. Smogunov, P. Umari, R. M. Wentzcovitch, QUANTUM ESPRESSO: A modular and open-source software project for quantum simulations of materials. *J. Phys. Condens. Matter.* **21**, 395502 (2009).

60. F. Neese, F. Wennmohs, U. Becker, C. Riplinger, The ORCA quantum chemistry program package. *J. Chem. Phys.* **152**, 224108 (2020).

61. P. H. Jacobse, MathemaTB: A Mathematica package for tight-binding calculations. *Comput. Phys. Commun.* **244**, 392–408 (2019).

62. T. Liu, Y. Ge, B. Sun, B. Fowler, H. Li, C. Nuckolls, S. Xiao, Synthesis, regioselective bromination, and functionalization of coronene tetracarboxydiimide. *J. Org. Chem.* **84**, 2713–2720 (2019).
